# Microbial modulation of bacoside A biosynthetic pathway and systemic defense mechanism in *Bacopa monnieri* under *Meloidogyne incognita* stress

**DOI:** 10.1038/srep41867

**Published:** 2017-02-03

**Authors:** Rupali Gupta, Akanksha Singh, Madhumita Srivastava, Vivek Singh, M. M. Gupta, Rakesh Pandey

**Affiliations:** 1 Department of Microbial Technology and Nematology, CSIR- Central Institute of Medicinal and Aromatic Plants, P.O. CIMAP, Lucknow 226015, India; 2 Academy of Scientific and Innovative Research, CSIR- Central Institute of Medicinal and Aromatic Plants Campus, Lucknow 226015, Uttar Pradesh, India; 3 Department of Analytical Chemistry, CSIR- Central Institute of Medicinal and Aromatic Plants, P.O. CIMAP, Lucknow 226015, India; 4 Department of Botany, Faculty of Science, Banaras Hindu University, Varanasi, 221005, India

## Abstract

Plant-associated beneficial microbes have been explored to fulfill the imperative function for plant health. However, their impact on the host secondary metabolite production and nematode disease management remains elusive. Our present work has shown that chitinolytic microbes *viz*., *Chitiniphilus* sp. MTN22 and *Streptomyces* sp. MTN14 singly as well as in combination modulated the biosynthetic pathway of bacoside A and systemic defense mechanism against *Meloidogyne incognita* in *Bacopa monnieri*. Interestingly, expression of bacoside biosynthetic pathway genes (3-Hydroxy-3-methylglutaryl coenzyme A reductase, mevalonate diphosphate decarboxylase, and squalene synthase) were upregulated in plants treated with the microbial combination in the presence as well as in absence of *M. incognita* stress. These microbes not only augmented bacoside A production (1.5 fold) but also strengthened host resistance *via* enhancement in chlorophyll a, defense enzymes and phenolic compounds like gallic acid, syringic acid, ferulic acid and cinnamic acid. Furthermore, elevated lignification and callose deposition in the microbial combination treated plants corroborate well with the above findings. Overall, the results provide novel insights into the underlying mechanisms of priming by beneficial microbes and underscore their capacity to trigger bacoside A production in *B. monnieri* under biotic stress.

Exploitation of beneficial microbes is well known to promote productivity, ameliorate nutrient supply and defend host from the various stresses. Among the wide range of microbes present in the soil, chitinolytic microbes have received extensive attention for their potential in plant growth and phyto-disease management. As a source of biocontrol, bacterial chitinases have been widely established for obstructing the growth of pathogen^1^,^2^. Chitinolytic microbes have been cited as antagonistic agents, which either act directly on nematodes egg shells or instigate increased plant resistance towards disease^3^. Therefore, we were interested in the development of a compatible microbial combination that effectively functions to protect disease mediated by plant parasitic nematode. Few studies have previously attempted to elucidate the relative quantitative and qualitative contributions of microbes to the production of secondary metabolites in plants^4^,^5^. However, the information regarding the mechanism involved is still unknown. Therefore, the use of microbes for boosting *in planta* secondary metabolite production under biotic stress could be a better and sustainable approach.

Utilization of medicinal plants for health consideration has become extremely popular with the indiscriminate use of synthetic drugs. *Bacopa monnieri* (L.) Pennell (family Scrophulariaceae), is second in the list of most essential Indian medicinal plants with ample number of therapeutically important bacosides^6^. Bacoside A is considered as a major active component known to have protective activities against morphine-induced cerebral toxicity, chemical-induced liver toxicity, and wound healing activity^7^,^8^. The remedial properties are because of the presence of bioactive saponins synthesized *via* mevalonic acid (MVA) pathway^9^. Globally, the requirement is met solely from the wild natural populations of *B. monnieri* resulting in its listing as a threatened plant^10^. In addition, the cultivation of *B. monnieri* faces stern damage owing to *Meloidogyne incognita* infestation^11^,^12^. To keep pace with the growing demand of this medicinal plant and considering its ability to grow under natural conditions, the present study was undertaken to develop strategies to improve yields of *B. monnieri* through beneficial microbial intervention. Previously, numerous methods have been employed to enhance plant immunity and the production of bioactive metabolites by the association between plant-beneficial microbes and their host plant (s), which suggests that the microbial partners could have a significant effect on host physiology^13^,^14^. However, till date, no information is available regarding the potential and possible mechanisms of the synergistic mode of microbes in the accumulation of secondary metabolites along with the modulation of the defense signaling pathway to endure biotic stress. Considering the above facts, efforts were made to examine the effect of synergistic chitinolytic microbial combination on induction of *in planta* bacoside A production and the possible mechanism adopted by *B. monnieri* plants under *M. incognita* stress.

## Results

### Chitinolytic microbes promote plant growth and effectively control *M. incognita*

**Figure 1:**
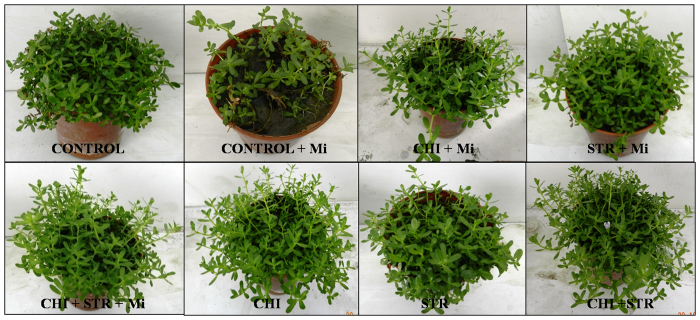
Plant growth of *B. monnieri* treated with chitinolytic microbes *viz*., *Chitiniphilus* sp. (CHI) and *Streptomyces* sp. (STR) applied singly as well as in combinations (with and without *M. incognita*). Control: untreated-uninoculated; Control + Mi: untreated *M. incognita-*inoculated.

**Figure 2:**
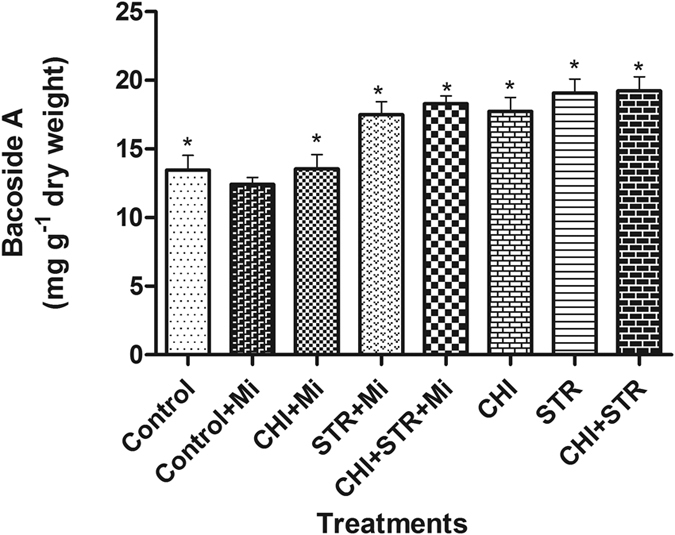
Effect of chitinolytic microbes *viz*., *Chitiniphilus* sp. (CHI) and *Streptomyces* sp. (STR) applied singly as well as in combinations (with and without *M. incognita*) on the bacoside A content in *B. monnieri*. Error bars indicate the standard error of six replicates. Asterisk indicate significant between treated and untreated pathogen-inoculated control (LSD test *P < 0.05).

To evaluate the effect of microbes on the growth and secondary metabolite production of *B. monnieri* plants under nematode stress, biomass, total bacoside content, and disease index were measured. Under greenhouse conditions, chitinolytic microbes *viz*., *Chitiniphilus* sp. MTN22 (KF699070) and *Streptomyces* sp. MTN14 (KF699062) alone and their combinational treatment showed a significant difference in growth parameters in the presence of *M. incognita* over untreated control (Fig. 1). After 5 weeks of nematode inoculation, the plants treated with chitinolytic microbes demonstrated their efficiency against *M. incognita* infestation by lessening the nematode number in the soil and roots as represented by the lower Rf values (Supplementary Table S1). *M. incognita* population in soil and roots were noticeably reduced (*P* ≤ 0.05) in alone *Chitiniphilus* (1.6 fold) and *Streptomyces* (1.5 fold) along with the combinational treatment (2.2 fold) inoculated with pathogen over untreated-*M. incognita* stressed plants. Among the various treatments, the most efficient control of *M. incognita* was observed in the microbial combination treated plants which (*P* ≤ 0.05) reduced the root galling index (RGI) by 2.8 fold, followed by alone *Chitiniphilus* sp. and *Streptomyces* sp. treatments which demonstrated reduction by 1.7 and 1.5 fold, respectively against untreated *M. incognita* control (Supplementary Table S1). The total bacoside content found in different treatments is given in Fig. 2. Plants treated with *Chitiniphilus* sp. and *Streptomyces* sp. without pathogen showed the greatest increment in bacoside A content (1.5 fold) than untreated pathogen-inoculated control (Fig. 2, Supplementary Fig. S1). Application of microbes noticeably increased bacoside A content (1.1 to 1.5 fold) as compared to untreated challenged control (Supplementary Table 1). In addition, there was a significant difference between the rhizospheric colonization of the microbial combination plants with and without pathogen over the single microbe treated plants (Supplementary Table S2). In the present experiment, chitinase activity showed a significant rise in dual microbes treated soil with respect to single treatments. The results showed that the chitinase activity was significantly raised in all the chitinolytic microbes treated soil. Further, analysis of the data revealed that higher activity was found in the microbial combination treated plants with pathogen followed by alone treatments compared to untreated *M. incognita* stressed plants (Supplementary Table S2).

Additionally, the nutrient uptake (NPK) status of *B. monnieri* was not much affected by the microbial inoculation (Supplementary Table S3). The maximum percentage of nitrogen content was noticed in the plants inoculated with dual microbes without (1.4 fold) and with (1.2 fold) pathogen, respectively while least nitrogen uptake was observed in the treatment of alone *Chitiniphilus* with pathogen (Supplementary Table S3). The phosphorus and potassium content were also found to be non significant among all the treatments in comparison to untreated pathogen-inoculated plants (Supplementary Table S3).

### The impact of chitinolytic microbes on the expression of various genes

**Figure 3:**
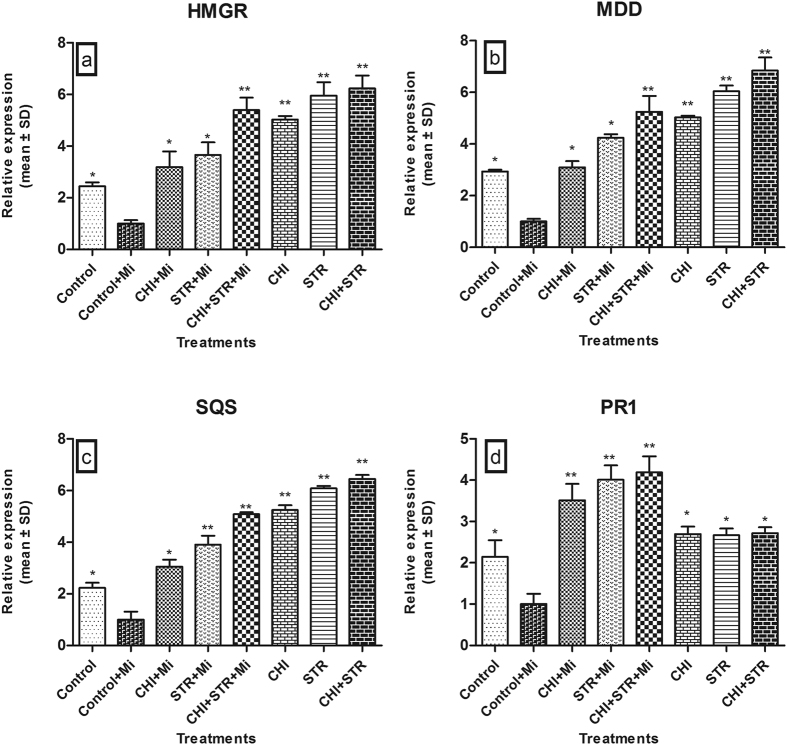
Expression analysis of various genes in *B. monnieri* as influenced by chitinolytic microbes *viz*., *Chitiniphilus* sp. (CHI) and *Streptomyces* sp. (STR) singly as well as in combinations (with and without *M. incognita*). Control: untreated-uninoculated; Control + Mi: untreated *M. incognita-*inoculated. Pathogenesis-related protein 1 (*PR1*), 3-hydroxy-3-methylglutaryl coenzyme A reductase (*HMGR*), mevalonate diphosphate decarboxylase (*MDD*), and squalene synthase (*SQS*) transcripts were amplified by qRT-PCR using gene-specific primers. Total RNA was isolated from the *B. monnieri* leaves after 5 weeks of *M. incognita* inoculation. The untreated *M. incognita* inoculated plant was used as a control. Results were normalized to 18S-rRNA (reference transcript). Relative expression was determined using the equation; 2^−ΔΔCt^. Data are mean ± standard error (n = 3 biological replicates) and asterisk indicate significant between treated and untreated pathogen-inoculated control (LSD test *P < 0.05; **P < 0.01).

We next examined whether microbial inoculation enhanced bacoside A production through the modulation of biosynthetic pathway gene expression. The results indicated that plants primed with chitinolytic microbes showed the highest level of 3-hydroxy-3-methylglutaryl coenzyme A reductase (*HMGR*), mevalonate diphosphate decarboxylase (*MDD*), and squalene synthase (*SQS*) transcription as compared to the control plants (Fig. 3). Expectedly, the microbial combination of *Chitiniphilus* sp. and *Streptomyces* sp. treated plants showed induced level of *HMGR* (6.2 fold) and *MDD* (6.8 fold) transcription compared to the untreated pathogen stressed control plants (Fig. 3a and b). Most interestingly, *SQS* expression was up-regulated and had the highest level (6.4 and 5.1 fold) in dual microbes treated plants with and without pathogen, respectively (Fig. 3c). Moreover, there was also a significant increment in the *PR1* (Pathogenesis-related protein 1) transcript levels in the plants treated with dual microbial treatment (4.2 fold) followed by *Streptomyces* (4.0 fold) and *Chitiniphilus* (3.5 fold) alone with pathogen (Fig. 3d).

### Influence of chitinolytic microbes on various enzymatic activities

**Figure 4:**
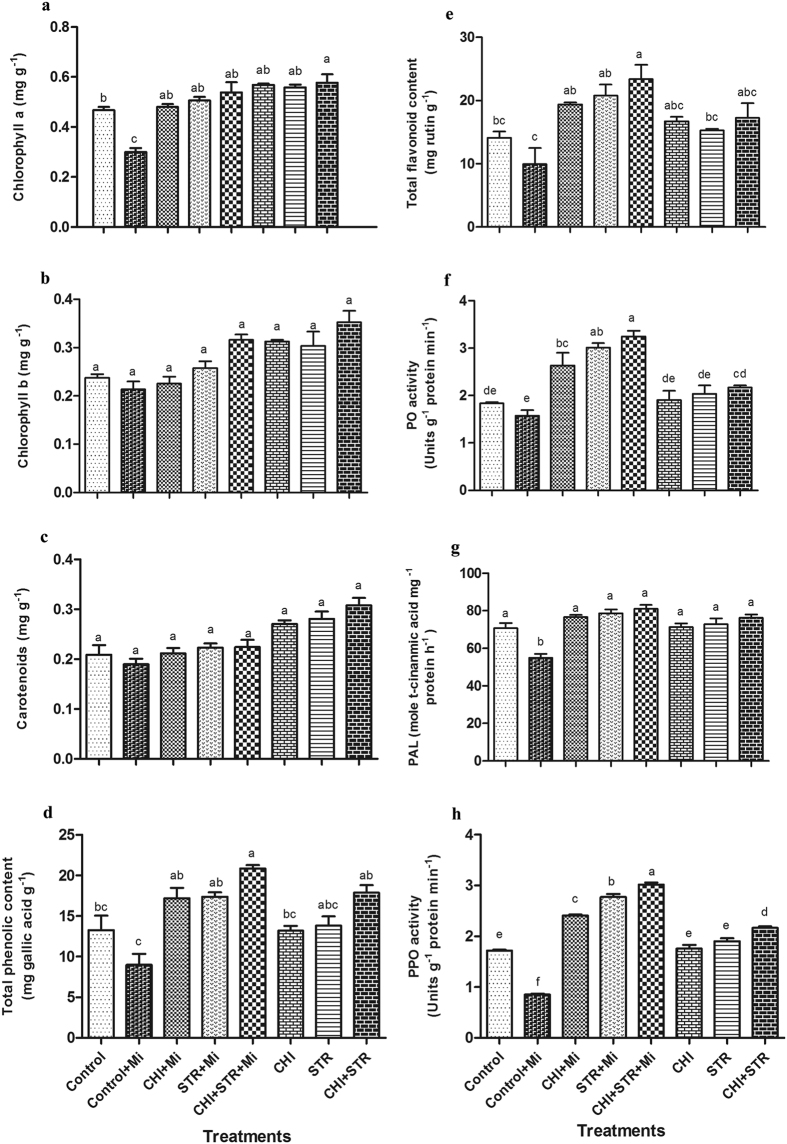
Estimation of chlorophyll, carotenoids and various defense-related enzymes in *B. monnieri* treated with chitinolytic microbes *viz*., *Chitiniphilus* sp. (CHI) and *Streptomyces* sp. (STR) singly as well as in combinations (with and without *M. incognita*). Control: untreated-uninoculated; Control + Mi: untreated *M. incognita-*inoculated. (**a**) Chlorophyll a; (**b**) Chlorophyll b; (**c**) Carotenoids; (**d**) Total phenolic content (TPC); (**e**) Total flavonoid content (TFC); (**f**) Peroxidase (PO) activity; (**g**) phenylalanine ammonia lyase (PAL) activity; and (**h**) polyphenol oxidase (PPO) activity. Error bars indicate the standard error of six replicates. Different letters indicate significant difference between treated and untreated pathogen-inoculated control (LSD test *P* < 0.05).

The effect of microbes on primary metabolism and defense enzymes of the plants under pathogenic stress compared with infected healthy and control plants was evaluated. Content of chlorophyll a was enhanced significantly whereas chlorophyll b and carotenoids were not affected by the microbial treatments (Fig. 4a,b and c). The increment in chlorophyll a was observed by 1.8 fold in the microbial combination treated plants followed by *Streptomyces* (1.7 fold) and *Chitiniphilus* (1.6 fold) with pathogen than untreated pathogen-inoculated control (Fig. 4a). In addition, higher levels of phenolic and flavonoid content were observed in dual (2.3 and 2.6 fold, respectively) followed by alone *Streptomyces* (1.9 and 2.1 fold, respectively) and *Chitiniphilus* (1.9 and 2.0 fold, respectively) treatments with pathogen (Fig. 4d and e). Furthermore, maximum peroxidase (PO), phenylalanine ammonia lyase (PAL) and polyphenol oxidase (PPO) activities were also recorded in the microbial combination (2.0, 1.5 and 3.5 fold, respectively) followed by *Streptomyces* (1.9, 1.4 and 3.3 fold, respectively) and *Chitiniphilus* (1.7, 1.4 and 2.8 fold, respectively) treatments in the presence of pathogen with respect to the pathogen-infected control plants (Fig. 4f,g and h).

### Phenolics compounds in leaves of *B. monnieri*

Considering that microbes act as an elicitor to enhance the defense parameters, we hypothesized that microbes might interfere with the phenylpropanoid and shikimate pathway. We thus investigated the effect of chitinolytic microbes on the phenolic status of the host plants. As expected, the variation in phenolics compounds was also found in the leaves of treated plants, under the influence of different microbial treatments (Table 1, Supplementary Fig. S2). The level of gallic acid varied from 5.3 to 22.8 μg g^−1^, syringic acid 0.6 to 5.1 μg g^−1^, ferulic acid 0.9 to 1.6 μg g^−1^, and cinnamic acid 0.2 to 2.5 μg g^−1^ dry weight (DW) in microbes treated plants (Table 1). The most effective treatment was dual combination of microbes where gallic acid accumulation was 1.4 fold, ferulic acid 1.8 fold, and cinnamic acid 2.5 fold higher as compared to the control treatment challenged only with the pathogen. However, maximum gallic acid was accumulated in dual microbial treatment in the presence of *M. incognita* (1.6 fold more) compared to untreated stressed plants. Further, syringic acid accumulation in leaves was highest in *Streptomyces* treatment inoculated with the pathogen. Interestingly, cinnamic acid and syringic acid were not detected in leaves of control treatment challenged with pathogen, *M. incognita* (Table 1, Supplementary Fig. S2).

### Lignin and callose deposition

**Figure 5:**
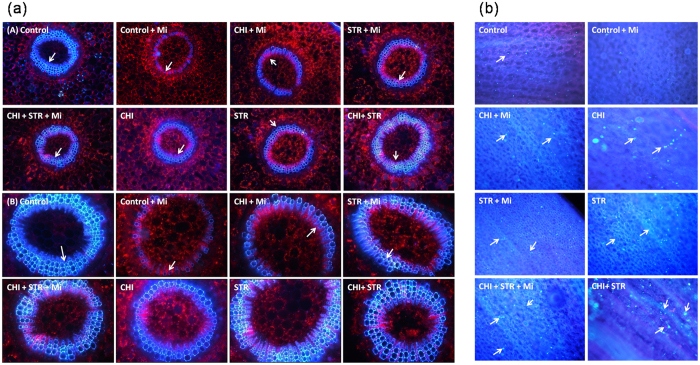
Lignification and callose deposition in *B. monnieri* treated with chitinolytic microbe’s *viz*., *Chitiniphilus* sp. (CHI) and *Streptomyces* sp. (STR) singly as well as in combinations (with and without *M. incognita*). Control: untreated-uninoculated; Control + Mi: untreated *M. incognita-*inoculated. (**aA** and **aB**) are magnification images at 5X (scale bar = 200 μm) and 10X (scale bar = 100 μm), respectively. Fluorescent microscope images; (**b**) aniline blue stained light microscope at 20X (scale bar = 200 μm).

To envisage the effect of microbial inoculation on the lignification and callose deposition, stem and leaves sections were visualized, respectively. *B. monnieri* plants uprooted from various treatments illustrated variation in the deposition of lignin when compared with untreated pathogen-inoculated plants (Fig. 5a). The maximum and uniform lignin deposition indicated as blue ring was found in the microbial combination with and without pathogen-treated plants followed by treatment having single microbial inoculation. In the control treatment challenged with pathogen, intermittent lignin deposits were observed (Fig. 5a). Additionally, callose deposition in leaves was found to be preferentially deposited in the interveinal region cells of host leaves as an intense blue-green fluorescence (Fig. 5b). Treatments with combination of microbes, along with alone *Chitiniphilus* and *Streptomyces* led to an increase in callose deposition in host leaves, with maximum enhancement in the microbial combination over untreated pathogen-inoculated treatment.

The results from the present study were further validated by principal component analysis. The formation of six different groups among the treatments: first forming only control, second cluster consisted of control challenged only with the pathogen, third having *Chitiniphilus* and *Streptomyces* with pathogen, fourth having dual microbes with pathogen, fifth having *Chitiniphilus* and *Streptomyces* treatments without pathogen and sixth having microbial combination treatment without pathogen. The microbial combination in the presence and absence of pathogen formed an individual group where all the morphological and physiological parameters along with bacoside content were enhanced. PCA denoted 83.2% of the total variance (PC1 denoted 57.4%, and PC2 denoted 25.8%) (Supplementary Fig. S3).

## Discussion

This study clearly demonstrated that when applied singly and in combinations, *Chitiniphilus* and *Streptomyces* act as antagonistic agents against *M. incognita* in terms of reducing galling index, reproduction factor and also ameliorated plant resistance along with the enhancement in secondary metabolites of plants. The enhanced disease protection could be possibly because of the chitinase producing microbes which might have effectively colonized the rhizospheric zone owing to the presence of easier nutrient sources in the soil. Also, the function of synergistic cooperation cannot be overlooked as our supposition is in conformity with the earlier findings, where the relationship between colonization and presence of chitinase in soil was highlighted^15^. The observations recorded are also in accordance with the previous studies of Krechel *et al*.^1^ and Oka *et al*.^16^ who demonstrated effective control of plant parasitic nematodes by chitinases producing rhizospheric microbes.

Along with the disease protection and plant growth promoting abilities of the beneficial microbes, induced synthesis of secondary metabolites is also reported^12^. In the present study, induction of bacoside A content *via* microbial application can thus be owed to the differential modulation in the expression of various genes involved in the bacoside pathway such as *HMGR, MDD* and *SQS*. It has been established that *B. monnieri* has two independent biosynthetic pathways; MVA occurring in the cytosol and the methyl-D-erythritol 4-phosphate (MEP) pathway in the plastid for triterpenoid saponins^17^. The MVA pathway initiates with the acetyl-coenzyme A, which condenses into the acetoacetyl-CoA by the catalyzing action of acetyl-CoA acetyltransferase. Acetyl-coenzyme A is converted to isopentenyl diphosphate (IPP) through MVA, and HMGR (HMG-CoA reductase) which finally supplies carbon for the bacoside biosynthesis^17^,^18^. In the present investigation, both the microbes upregulated the expression of *HMGR*, a post-transcriptionally as well as post-translationally regulated gene responsible for catalyzing the conversion of HMG-CoA to mevalonate^19^. In a previous study, the role of *HMGR* in isoprene biosynthesis was identified as loss of function of *HMGR1* lead to dwarfism and early senescence in *Arabidopsis*^20^.

As a key regulatory enzyme of MVA pathway, mevalonate diphosphate decarboxylase (*MDD*) catalyzes the decarboxylation of the mevalonate 5- diphosphate (MVAPP) to the isopentenyl IPP, accompanied by the hydrolysis of ATP^21^. Likewise, squalene synthase (SQS) is a bifunctional enzyme which catalyzes reaction at an important regulatory point that controls carbon flux into sterol and triterpenoid biosynthetic pathway^22^. Upregulation of *HMGR, MDD* and *SQS* expression in microbes-inoculated plants may be the explanation for the higher bacoside A production. Our elucidation is also supported by the earlier findings where application of microbes over expressing biosynthetic genes, had elevated level of secondary metabolites as compared to the wild-type plants^12^,^23^. The findings further advocated that the bacoside synthesis is preferentially favored, by the significant expression of gene transcripts related to this pathway in chitinolytic microbes treated plants over the control. Augmentation in the bacoside content could be the result of upregulation of *HMGR, MDD* and *SQS* genes which was further substantiated by the high-performance liquid chromatography (HPLC) analysis. Thus, our findings strongly suggest that the applications of beneficial microbes are able to promote the biosynthetic potential of the plant for key secondary metabolites.

In the present investigation, the microbes not only reduced *M. incognita* infestation but also systemically provoked resistance without affecting the photosynthetic efficiency in terms of chlorophyll b and carotenoid content of the host plant. The principal groups of metabolites in plants known for their function in host plant resistance are phenolics and flavonoids. Collectively, improved PPO, PO, and PAL activities which are important defense enzymes of plants can be positively correlated with the bolstered up plant systemic resistance against the pathogen. In addition to the above defense enzymes, lignin plays an imperative role in protecting plants against pathogens and provides structural integrity to the host cell wall during the itinerary of normal tissue development^24^. Since, our results confirmed higher PPO activity in the dual microbe treated plants which is reported to catalyze phenols oxidation and finally lignification, we next studied lignin deposition pattern in the different treatments. Observations revealed disparity in lignin deposition in *B. monnieri* which could be accredited to the efficacy of the chitinolyitc microbes to prompt the lignification process.

Recently, there has been a terrific development concerning the utilization of chitinolytic microbes to enhance plant immunity^25^. The results demonstrated evidently that the production of phenolic compounds had a positive connection with disease diminution as less severity was observed in the treatments having high concentration of phenolic compounds. Furthermore, induced PAL activity which is a precursor of phenylpropanoid pathway may be the reason of elevated phenolic level in the dual microbe treatment. Presence of phenolic acids mainly gallic, ferulic, syringic and cinnamic acids in leaves indicated that the defense response induced by chitinolytic microbes is altered accordingly to the physiological state of the plant. The enhanced defense response also supports the reason behind improved plant growth in chitinolytic microbe treatments. In addition, the consistent increase of gallic acid in leaves of plants demonstrates that this compound, occurs constitutively, and its amount is modulated by the application of beneficial microbes. Elevated gallic acid level which is mainly responsible for crosslinking of suberin and lignin as well as other polyphenolic barriers in cell wall formed after pathogen infection might also be the reason for the presence of more lignified cells in the dual microbe treatment in comparison to other treatments^26^. Further, intensification in the defense response of plants was further validated by the enhanced callose deposition responsible for strengthening of the cell wall.

Ferulic and cinnamic acids formed through the shikimic acid pathway are well known antifungal and potent antioxidants phenolics^27^. A high amount of ferulic acid in the plants undeniably supports their function in reducing pathogenic stress. Sarma *et al*.^28^ and Takenaka *et al*.^29^ reported that the increased levels of ferulic acid in the host plants with beneficial microbes played a major role in reducing the biotic stress. Better disease protection efficiency of the *Chitiniphilus* and *Streptomyces* strains could also be possibly by the accumulation of antimicrobial cinnamic acid and syringic acid in treated plants challenged with the pathogen. However, at this stage possibility of participation of some other more complex molecular alterations in plants after the application of chitinolytic microbes resulting in the activation of defense responses through synthesis of certain enzymes linked with host resistance cannot be ruled out. Overall, the earlier findings of less mortality in host plants against various pathogens after application of beneficial microbes^28^,^30^ was further proved in the present study.

**Figure 6:**
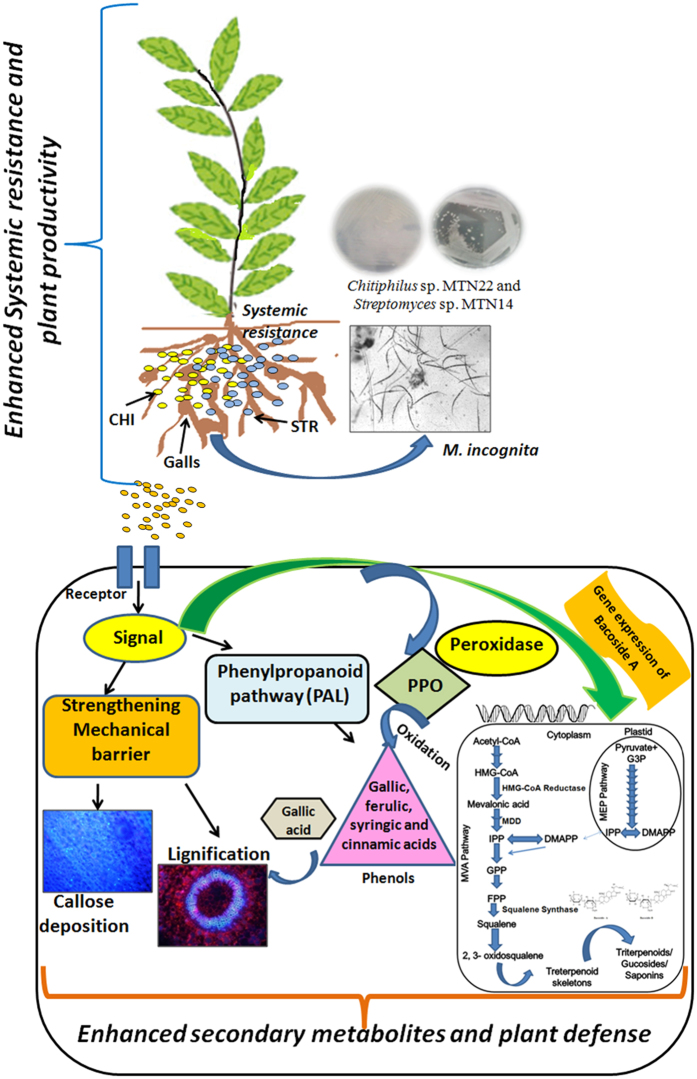
Mechanistic figure representing chitinolytic microbes mediated secondary metabolite enhancement and defense responses in *B. monnieri* against biotic stress.

Results of the present study suggest that the presence of *Chitiniphilus* and *Streptomyces* in the rhizosphere of plant probably plays an important role in promoting the growth of the host plant by increasing chlorophyll a, defense enzymes, lignification, callose deposition along with increased bacoside A accumulation and disease suppression (Fig. 6). The absence of these microbes resulted in reduced growth and secondary metabolite production which further verified our above observations. Our study strongly highlights that the presence of chitinolytic microbes is able to promote the biosynthetic potential of the plant for some key secondary metabolites. Both *Chitiniphilus* and *Streptomyces* enhanced bacoside A content by modulating the expression of genes involved in biosynthesis pathway. For future applications, we suggest chitinase based formulations using chitinolytic microbes (that enhance plant growth rate, biomass and bacoside content) under natural conditions. Such microbial combination may not only provide a middle course by providing high productivity and protection coupled with high bacoside A but will also play a key role in dropping the cost of the expensive bacoside.

## Methods

### Microbes and pathogen inoculums

*Chitiniphilus* sp. and *Streptomyces* sp. were individually inoculated in Luria Bertani (LB) and Glucose Yeast Malt (GYM) broth, respectively and incubated at 30 ± 2 °C on a rotary shaker (120 rpm) for 1 day and 7 days, respectively for mass production. The centrifugation was done and the pellet density was maintained in 0.85% saline to 1 × 10^8^ colony forming units (CFU) mL^−1^. We previously isolated *Chitiniphilus* and *Streptomyces* with high nematicidal and chitinase activity against *M. incognita*^31^.

*M. incognita* culture was isolated from *Solanum melongena* L. plants grown in greenhouse conditions. The intact plants were uprooted and egg masses were extracted using an extraction procedure^32^. The J2 juveniles were obtained from extracted eggs that were continuously oxygenated for 1 week using an aquarium pump^33^.

### Treatment of plants with *Chitiniphilus* sp. and *Streptomyces* sp.

The *B. monnieri* (cv. CIM-Jagriti) runners of even length (6–8 cm) bearing at least 3–4 nodes along with roots were transplanted to the experimental pots containing 0.5 kg of sterilized sandy soil (pH 7.5). The pots consisted of autoclaved alkaline (pH 7.5, EC 0.30 dSm^−1^) sandy loam soil: vermicompost (3:1 w/w) and contained available nitrogen (142.00 kg ha^−1^), organic carbon (4.40 g kg^−1^), phosphorus (9.21 kg ha^−1^) and potassium (95.00 kg ha^−1^). Following treatments were used in the experiment: *Chitiniphilus* sp., *Streptomyces* sp. and microbial combination with pathogen along with two controls: untreated with and without pathogen. The plants were maintained in a greenhouse and all the sets were arranged in a completely randomized design with nine replicates. A separate set of treatments having chitinolytic microbes only (without pathogen) was also maintained. The microbial suspensions were applied at a dose of 10 mL per pot. In the combinational treatment, cell suspension of the two strains was mixed in equal ratio and vortexed to obtain homogenous mixture. Freshly hatched J2 juveniles of *M. incognita* (1000 J2) were inoculated in each of the pots after one week of microbial inoculation. The experiment was repeated twice and the results were collected 5 weeks after *M. incognita* inoculation. The pathogen population density was estimated in the soil by Cobb’s sieving and decanting technique followed by Baermann funnel^33^. The nematode and egg populations in the plant root system were analyzed by macerating 2 g aliquots of root tissues and the nematode population was calculated through reproduction factor Rf = Pf (final *M. incognita* population)/Pi (initial *M. incognita* population)^34^. The severity of root galling index (RGI) in root knot infested seedlings was assessed according to Oka *et al*.^35^ on 0–10 scale. The inoculated chitinolytic microbes density in the soil was calculated as colony forming unit (CFU) g^−1^. The chitinase activity was also measured in the rhizospheric soil from each treatment pot^−1^ by using 4-methylumbelliferyl-(GlcNAc)^2^ [4MU − (GlcNAc)^2^] (Sigma) according to Metcalfe *et al*.^36^. Nutrient uptake in dry matter of various treatments was determined based on NPK content by Jackson *et al*.^37^.

### Extraction and estimation of bacoside A content

The leaves of different treatments were shade dried and methanolic extract was prepared from 100 mg of each sample. The combined methanolic extract was concentrated under vacuum for analysis. The process was repeated six times. The concentration of bacoside-A was measured by HPLC^38^.

### Transcription analysis

Total RNA was isolated by trizol (Invitrogen) method and cDNA was reverse-transcribed with random primers kit (Thermo Fisher Scientific, United States). A gene 18S-rRNA was taken as the internal control. The primer sets for the genes *PR1, HMGR*, and *MDD* except *SQS* were designed using Primer 3 version 0.4.0. The effectiveness of the designed primer sets was assessed by measuring the PCR amplicons length in agarose gel. All the primers involved in the experiment are mentioned in Supplementary Table S4. The following reactions were carried out in the qRT-PCR for each of the target genes: 1 cycle of 10 min at 94 °C, followed by 40 cycles of 30 s at 94 °C, 30 s at 55 °C, and 30 s at 72 °C and at the end of the PCR cycles the amplicon dissociation curve was recorded. The reactions were repeated twice in triplicate. The mean expression of various genes was analyzed by the (2^−ΔΔCt^) comparative CT method^39^.

### Phenolic compounds in the leaves of *B. monnieri*

For HPLC analysis, 100 mg of leaves was dried and extracted with 50% methanol (10 mL). The solvent was removed using rotary evaporator and the residue was dissolved in HPLC grade methanol and subjected to HPLC for the detection of specific phenolic compounds^40^. The process was repeated six times. The data (μg g^−1^ DW) was calculated by comparing the peak areas (*λ*_max_ 254 nm) of the treated samples with their respective standards (Class VP series software, Shimadzu, Japan).

### Enzymatic assays

The seedlings from each treatment were randomly uprooted and immediately frozen using liquid nitrogen. The root material (1 g) was homogenized using 10 mM sodium phosphate buffer (pH 6.0) with polyvinylpolypyrrolidone (1% w/v), phenylmethylsulfonyl fluoride (0.3 mM), and EDTA (1 mM) at 4 °C and was filtered through nylon filter. The centrifugation was done at 10,000 × *g* for 15 min and the crude extract was used for enzymatic activities^41^. All the assays were repeated six times.

Determination of chlorophyll and carotenoid content from powdered leaves tissue was done according to Arnon^42^ and Lichtenthaler *et al*.^43^, respectively. The powdered leaves (100 mg) samples were extracted with CH_3_OH:H_2_O (9:1, v/v) solution. The centrifugation was done at 8,000 × *g* for 5 min and absorbance was calculated at 663, 645, 480 and 510 nm. The total phenolic content (TPC) was expressed as mg gallic acid equivalent per g of dried sample^44^. The methanolic extract of leaves tissue (1 mL) was mixed with folin ciocalteu reagent (0.5 mL) along with sodium carbonate solution (7%; 2.5 mL) and incubated at room temperature for 2 h. The absorbance was recorded at 765 nm. Total flavonoid content (TFC) was calculated using rutin (mg g^−1^) as standard^45^. A 15% sodium nitrite solution (0.15 mL) was added to extract (0.5 mL). The 4% NaOH (2 mL) was added after 6 min of incubation and the volume of reaction mixture was made upto 5 mL which was further left for incubation for 15 min. The absorbance was calculated at 510 nm. PO activity was determined as unit mg^−1^ protein per min^−1^ at 420 nm^46^. The reaction mixture contained 100 μL of the diluted extract (10 fold) with 1.5 mL pyrogallol (0.05 mol L^−1^) and 0.5 mL H_2_O_2_ (1% v/v). The alteration in the absorbance was measured after 30 s intervals for 3 min at 420 nm. PAL activity was measured by adding enzyme extract (100 μL) in L-phenylalanine (6 μM; 900 μL) and Tris-HCl buffer solution as mentioned by Chen *et al*.^46^ and was denoted in mole trans-cinnamic acid g^−1^ protein h^−1^. The tubes were incubated for 70 min at room temperature in a water bath and the absorbance was measured spectrophotometrically at 290 nm. PPO activity was calculated by mixing enzyme extract (100 μL) in 700 μL of homogenization buffer according to Gauillard *et al*.^47^ and was measured as unit mg^−1^ protein per min^−1^. After the addition of catechol (0.2 M), the change in absorbency was measured for 1 min at 405 nm. For control, the reaction mixture without enzyme extract was also maintained.

### Lignification and callose deposition

*B. monnieri* plants were harvested 5 weeks after the inoculation of pathogen, *M. incognita*. For imaging lignification pattern, the transverse sections of stem were examined under fluorescent microscope DMI 3000 B (Leica, Wetzlar, Germany) with a wide range of wavelengths (250–700 nm) to induce autofluorescence^13^. For callose detection, leaves were boiled in ethanol and placed in 0.01% aniline blue for 1 h. The samples were examined for the presence of cell wall deposition using a UV epifluorescence microscope^48^. Four biological repeats were performed for each test and at least 10 leaves were collected for the inspection from multiple seedlings in each experiment.

### Statistical analysis

The significant differences between the various treatments and infected control were analyzed by LSD (least significant difference; *P* ≤ 0.05) using SPSS package (SPSS V16.0, SPSS Inc., Chicago, IL).

## Additional Information

**How to cite this article**: Gupta, R. *et al*. Microbial modulation of bacoside A biosynthetic pathway and systemic defense mechanism in *Bacopa monnieri* under *Meloidogyne incognita* stress. *Sci. Rep.* **7**, 41867; doi: 10.1038/srep41867 (2017).

**Publisher's note:** Springer Nature remains neutral with regard to jurisdictional claims in published maps and institutional affiliations.

## Supplementary Material

Supplementary Information

## Figures and Tables

**Table 1:** Effect of chitinolytic microbes *viz*., *Chitiniphilus* sp. (CHI) and *Streptomyces* sp. (STR) singly as well as in combinations (with and without *M. incognita*) on phenolics content in *B. monnieri*.

Treatments	Phenolics			
	Gallic acid	Syringic acid	Ferulic acid	Cinnamic acid
Control	5.34 ± 0.58^d^	3.66 ± 0.18^b^	0.96 ± 0.23^b^	ND
Control with Mi	14.64 ± 0.29^c^	ND	0.89 ± 0.06^b^	ND
CHI + Mi	21.13 ± 0.58^b^	2.02 ± 0.29^c^	1.10 ± 0.06^ab^	1.22 ± 0.07^b^
STR + Mi	21.25 ± 0.18^ab^	5.09 ± 0.01^a^	ND	2.87 ± 0.23^a^
CHI + STR + Mi	22.79 ± 0.11^a^	0.56 ± 0.02^d^	ND	0.23 ± 0.09 ^cd^
CHI	14.61 ± 0.05^c^	ND	1.26 ± 0.22^ab^	ND
STR	14.41 ± 0.20^c^	1.87 ± 0.12^c^	0.91 ± 0.06^b^	0.69 ± 0.29^bc^
CHI + STR	20.28 ± 0.09^b^	ND	1.64 ± 0.15^a^	2.51 ± 0.06^a^

Control: untreated-uninoculated; Control + Mi: untreated *M. incognita-*inoculated. Results represent mean ± standard error of six replicates. Different letters indicate significant difference between treated and untreated pathogen-inoculated control (LSD test *P* < 0.05). ND not determined.
