# Supplementary material for: Microbial modulation of bacoside A biosynthetic pathway and systemic defense mechanism in Bacopa monnieri under Meloidogyne incognita stress
Source: Sci Rep. 2017 Feb 3;7:41867. doi: 10.1038/srep41867 (PMC5291102; doi:10.1038/srep41867)
Supplement: Supplementary Information [file srep41867-s1.doc]

**Supplementary Material**

**Microbial modulation of bacoside A biosynthetic pathway and systemic defense mechanism in *Bacopa monnieri* under Meloidogyne *incognita* stress**

**Rupali Gupta, Akanksha Singh, Madhumita Srivastava, Vivek Singh, M.M. Gupta and Rakesh Pandey**

**Supplementary Table S1** **Effect of** **chitinolytic microbe’s *viz.,* *Chitiniphilus* sp. (CHI) and *Streptomyces* sp. (STR) singly as well as in combinations (with and without *M. incognita*) on population density, reproduction factor and root gall index of *B. monnieri.*** Control: untreated-uninoculated; Control + Mi: untreated *M. incognita-*inoculated. Results represent mean ± standard error of six replicates. Different letters indicate significant difference between treated and untreated pathogen-inoculated control (LSD test *P* < 0.05).

| **Treatments** | **Fresh biomass (g)** | **Dry biomass (g)** | **Nematode population**  **Density/pot**  **(soil + root)** | **Reproduction factor (Rf)** | **Root Galling Index (RGI)**  **(0-10)** |
| --- | --- | --- | --- | --- | --- |
| Control | 27.14 ± 1.73**^de^** | 6.14 ± 0.59**^ab^** | 0.00 ± 0.00**^d^** | 0.00 ± 0.00**^c^** | 0.00 ± 0.00**^d^** |
| Control+Mi | 23.33 ± 1.60**^e^** | 4.42 ± 0.56**^b^** | 4578.00 ± 64.08**^a^** | 4.58 ± 0.93**^a^** | 8.5 ± 0.87**^a^** |
| CHI+Mi | 35.14 ± 1.91**^cd^** | 7.12 ± 1.63**^ab^** | 2869.00 ± 40.41**^b^** | 2.87 ± 0.22**^ab^** | 5.0 ± 0.58**^bc^** |
| STR+Mi | 38.21 ± 1.12**^bc^** | 7.92 ± 1.27**^ab^** | 3048.00 ± 18.47**^b^** | 3.04 ± 0.59**^ab^** | 5.5 ± 0.58**^b^** |
| CHI+STR+Mi | 41.71 ± 1.88**^abc^** | 8.14 ± 0.75**^ab^** | 2074.00 ± 18.41**^c^** | 2.07 ± 0.47**^bc^** | 3.0 ± 0.29**^c^** |
| CHI | 38.14 ± 1.41**^bc^** | 8.35 ± 0.95**^ab^** | 0.00 ± 0.00**^d^** | 0.00 ± 0.00**^c^** | 0.00 ± 0.00**^d^** |
| STR | 44.14 ± 1.49**^ab^** | 8.71 ± 0.51**^ab^** | 0.00 ± 0.00**^d^** | 0.00 ± 0.00**^c^** | 0.00 ± 0.00**^d^** |
| CHI+STR | 46.92 ± 1.81**^a^** | 9.14 ± 1.16**^a^** | 0.00 ± 0.00**^d^** | 0.00 ± 0.00**^c^** | 0.00 ± 0.00**^d^** |

**Supplementary Fig. S1: HPLC chromatogram of bacoside A content of *B. monnieri* treated with chitinolytic microbes *viz.,* *Chitiniphilus* sp. (CHI) and *Streptomyces* sp. (STR) singly as well as in combinations (with and without *M. incognita*).** Control: untreated-uninoculated; Control + Mi: untreated *M. incognita-*inoculated. All the samples were collected 5 weeks after challenge with *M. incognita.*


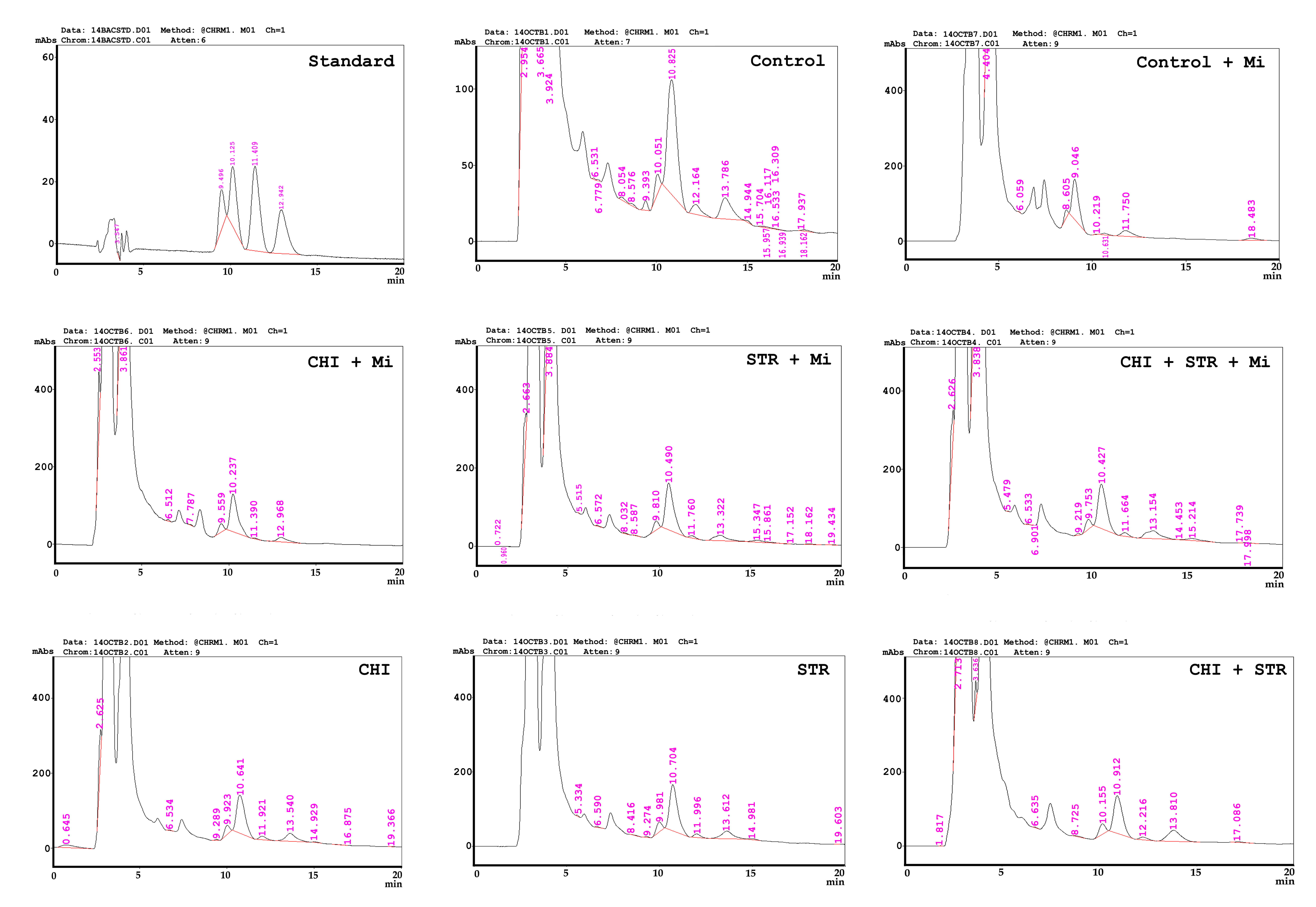


**Supplementary Table S2** **Effect of** **chitinolytic microbes *viz.,* *Chitiniphilus* sp. (CHI) and *Streptomyces* sp. (STR) singly as well as in combinations (with and without *M. incognita*) on the rhizospheric colonization (CFU g^-1^) and chitinase activity of *B. monnieri.*** Control: untreated-uninoculated; Control + Mi: untreated *M. incognita-*inoculated. Results represent mean ± standard error of six replicates. Different letters indicate significant difference between treated and untreated pathogen-inoculated control (LSD test *P* < 0.05).

| **Treatments** | **CFU (10^4^ g^-1^ of soil)** | | **Chitinase activity (µmoles 4-MUF Hydrolyse ml^-1^ g^-1^ of soil)** |
| --- | --- | --- | --- |
|  | **CHI** | **STR** |  |
| Control | - | - | 0.13 ± 0.03**^c^** |
| Control+Mi | - | - | 0.12 ± 0.01**^c^** |
| CHI+Mi | 3.25 ± 0.35**^ab^** | - | 4.27 ± 0.18**^b^** |
| STR+Mi | - | 4.74 ± 0.17**^b^** | 5.20 ± 0.07**^ab^** |
| CHI+STR+Mi | 3.75 ± 1.31**^ab^** | 5.75 ± 0.16**^a^** | 5.98 ± 0.69**^a^** |
| CHI | 4.33 ± 0.27**^b^** | - | 0.98 ± 0.05**^c^** |
| STR | - | 4.85 ± 0.48**^a^** | 1.27 ± 0.41**^c^** |
| CHI+STR | 4.96 ± 1.84**^b^** | 5.25 ± 1.32**^a^** | 1.58 ± 0.49**^c^** |

**Supplementary Table S3 Effect of chitinolytic microbes *viz.,* *Chitiniphilus* sp. (CHI) and *Streptomyces* sp. (STR) singly as well as in combinations (with and without *M. incognita*) on the nutrient uptake of *B. monnieri.*** Control: untreated-uninoculated; Control + Mi: untreated *M. incognita-*inoculated. Results represent mean ± standard error of six replicates. Different letters indicate significant difference between treated and untreated pathogen-inoculated control (LSD test *P* < 0.05).

| **Treatments** | **Nitrogen (%)** | **Phosphorus (%)** | **Potassium (%)** |
| --- | --- | --- | --- |
| Control | 1.48 ± 0.20**^a^** | 0.27 ± 0.04**^a^** | 1.44 ± 0.11**^a^** |
| Control+Mi | 1.35 ± 0.08**^a^** | 0.28 ± 0.10**^a^** | 1.29 ± 0.09**^a^** |
| CHI+Mi | 1.48 ± 0.09**^a^** | 0.30 ± 0.06**^a^** | 1.51 ± 0.17**^a^** |
| STR+Mi | 1.48 ± 0.22**^a^** | 0.28 ± 0.07**^a^** | 1.29 ± 0.09**^a^** |
| CHI+STR+Mi | 1.64 ± 0.15**^a^** | 0.30 ± 0.09**^a^** | 1.66 ± 0.14**^a^** |
| CHI | 1.59 ± 0.07**^a^** | 0.29 ± 0.05**^a^** | 1.47 ± 0.21**^a^** |
| STR | 1.86 ± 0.05**^a^** | 0.28 ± 0.01**^a^** | 1.43 ± 0.14**^a^** |
| CHI+STR | 1.89 ± 0.25**^a^** | 0.30 ± 0.06**^a^** | 1.43 ± 0.30**^a^** |

**Supplementary Fig. S2: HPLC analysis of ethyl acetate fractions of *B. monnieri* treated with chitinolytic microbes *viz.,* *Chitiniphilus* sp. (CHI) and *Streptomyces* sp. (STR) singly as well as in combinations (with and without *M. incognita*).** Control: untreated-uninoculated; Control + Mi: untreated *M. incognita-*inoculated. All the treatments were collected 5 weeks after challenge with *M. incognita* and a–d represents: (a) gallic acid, (b) syringic acid, (c) ferulic acid, and (d) cinnamic acid. In the figure, Y-axis of each chromatogram represents absorbance unit, whereas, x-axis represents retention time in min.


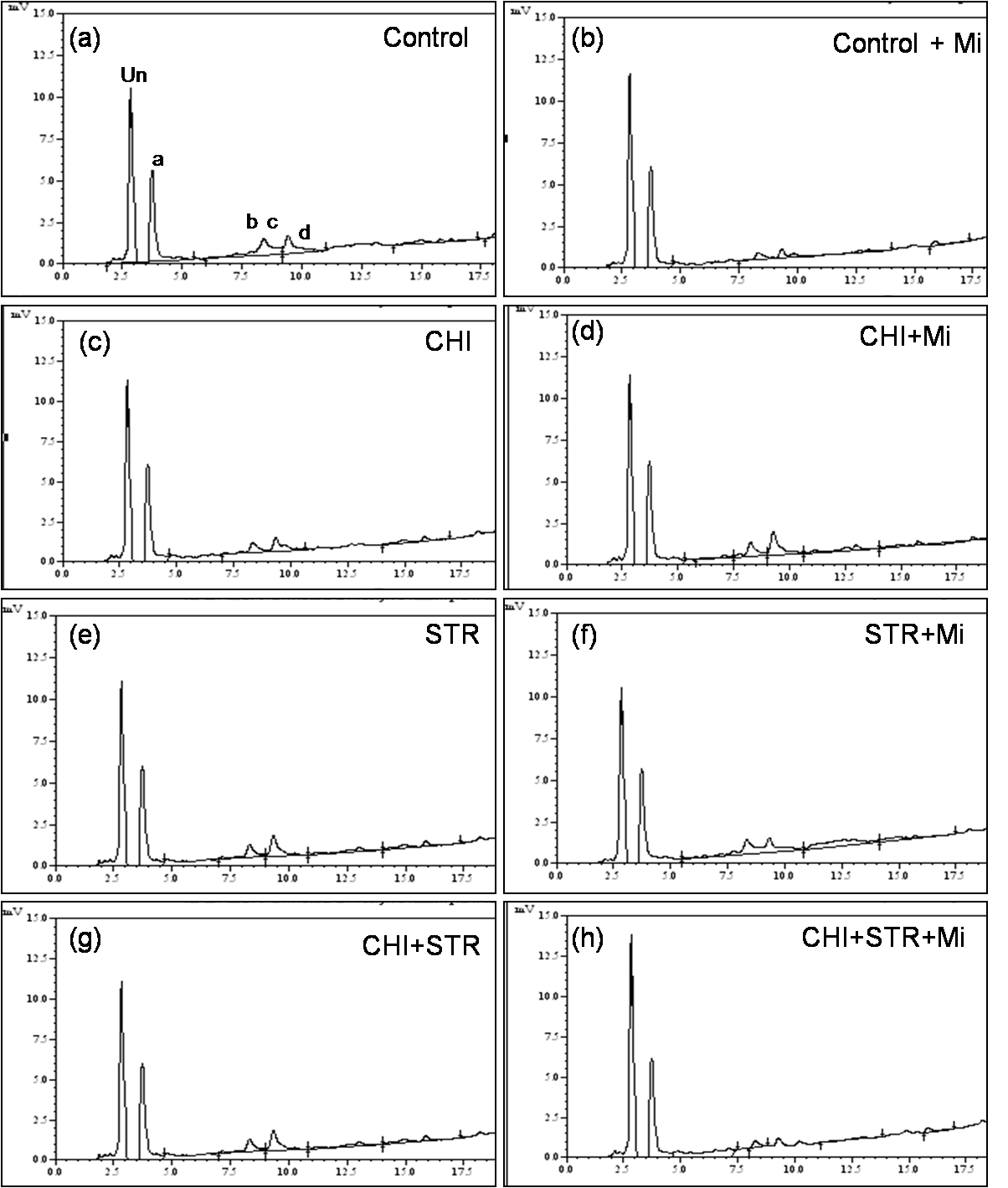


**Supplementary Fig. S3: Principal component analysis (PCA) of various physiological, biochemical and disease index parameters analyzed in *B. monnieri* treated with chitinolytic microbe’s *viz.,* *Chitiniphilus* sp. (CHI) and *Streptomyces* sp. (STR) singly as well as in combinations (with and without *M. incognita*).** Control: untreated-uninoculated; Control + Mi: untreated *M. incognita-*inoculated.

**
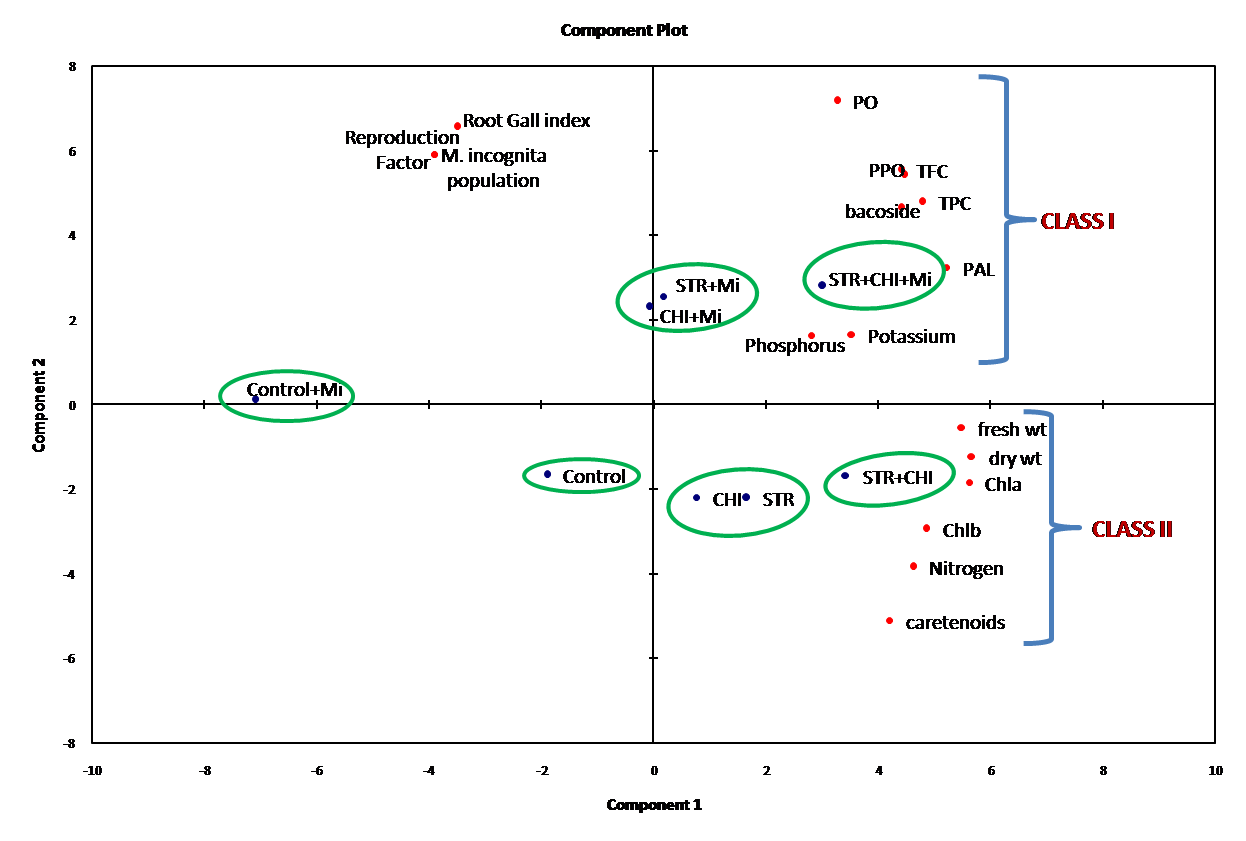
**

**Supplementary Table S4 List of primers used for Real time PCR (RT-PCR) in this study.**

| **Gene** | **Sequence** | **Reference** |
| --- | --- | --- |
| 18S-rRNA | Forward: GCACGCGCGCTACACCGAAG  Reverse: GTCTGTACAAAGGGCAGGGACG | [17] |
| *PR1*  (Pathogenesis-related protein 1) | Forward: GAGCACTCAGATGGACCGTA  Reverse: CGCCACACAACTTGCGTATA | In this study |
| *SQS* (Squalene synthase) | Forward: TTGCGAGATCCAGCGATCTTTCGT Reverse: TAGCTGTGAGCCCACGTCTCATTT | [17] |
| *HMGR*  (3-Hydroxy-3-methylglutaryl coenzyme A reductase) | Forward: AGCGGCTGTGAATTGGATTG  Reverse: GCGATATACACAGCCGACAC | In this study |
| *MDD*  (Mevalonate diphosphate decarboxylase) | Forward: AGGTGCCTCTTTCTGGAGTC  Reverse: CGGCAGCAGTAGGGAAATTG | In this study |
